# Comparison of methods for the enumeration of enterohemorrhagic *Escherichia coli* from veal hides and carcasses

**DOI:** 10.3389/fmicb.2015.01062

**Published:** 2015-09-29

**Authors:** Brandon E. Luedtke, Joseph M. Bosilevac

**Affiliations:** U. S. Department of Agriculture, Agricultural Research Service, Roman L. Hruska U. S. Meat Animal Research CenterClay Center, NE, USA

**Keywords:** veal, total EHEC, multiplex qPCR, MPN, dPCR

## Abstract

The increased association of enterohemorrhagic *Escherichia coli* (EHEC) with veal calves has led the United States Department of Agriculture Food Safety and Inspection Service to report results of veal meat contaminated with the Top 7 serogroups separately from beef cattle. However, detection methods that can also provide concentration for determining the prevalence and abundance of EHEC associated with veal are lacking. Here we compared the ability of qPCR and a molecular based most probable number assay (MPN) to detect and enumerate EHEC from veal hides at the abattoir and the resulting pre-intervention carcasses. In addition, digital PCR (dPCR) was used to analyze select samples. The qPCR assay was able to enumerate total EHEC in 32% of the hide samples with a range of approximately 34 to 91,412 CFUs/100 cm^2^ (95% CI 4-113,460 CFUs/100 cm^2^). Using the MPN assay, total EHEC was enumerable in 48% of the hide samples and ranged from approximately 1 to greater than 17,022 CFUs/100 cm^2^ (95% CI 0.4–72,000 CFUs/100 cm^2^). The carcass samples had lower amounts of EHEC with a range of approximately 4–275 CFUs/100 cm^2^ (95% CI 3–953 CFUs/100 cm^2^) from 17% of samples with an enumerable amount of EHEC by qPCR. For the MPN assay, the carcass samples ranged from 0.1 to 1 CFUs/100 cm^2^ (95% CI 0.02–4 CFUs/100 cm^2^) from 29% of the samples. The correlation coefficient between the qPCR and MPN enumeration methods indicated a moderate relation (*R*^2^ = 0.39) for the hide samples while the carcass samples had no relation (*R*^2^ = 0.002), which was likely due to most samples having an amount of total EHEC below the reliable limit of quantification for qPCR. Interestingly, after enrichment, 81% of the hide samples and 94% of the carcass samples had a detectable amount of total EHEC by qPCR. From our analysis, the MPN assay provided a higher percentage of enumerable hide and carcass samples, however determining an appropriate dilution range and the limited throughput offer additional challenges.

## Introduction

Shiga-toxin producing *Escherichia coli* (STEC) are an increasing concern in relation to food safety. The United States Department of Agriculture (USDA) Food Safety and Inspection Service (FSIS) has identified the pathogenic strains of the serogroups O26, O45, O103, O111, O121, and O145 (Top 6) in addition to O157 as being adulterants in non-intact beef (Almanza, 2011). However, emerging STEC serogroups pose a threat to human health with emphasis on the STEC subgroup that comprises the enterohemorrhagic *E. coli* (EHEC). Approximately 20% of human illnesses caused by a non-O157 EHEC were attributed to a serogroup not identified by FSIS (Brooks et al., 2005; Gould, 2009). The EHEC serogroups mostly cause the severest form of disease and can result in hemorrhagic colitis and/or hemolytic uremic syndrome primarily in children under 10 and the elderly (Goldwater and Bettelheim, 2012). In the environment, cattle act as the primary reservoir for EHEC and facilitate the transmission of the bacteria through the release of contaminated feces. Moreover, during the harvesting of cattle, EHEC can contaminate the carcass via the transfer of feces from the animal hide (Elder et al., 2000; Monaghan et al., 2012).

Recently, FSIS has placed interest in the increased association of the adulterant EHEC with veal products compared to beef (United States Department of Agriculture and Food Safety and Inspection Service, 2012) and has implicated a hide to carcass transmission as the primary mode of contamination (United States Department of Agriculture, and Food Safety and Inspection Service, 2013). Indeed, among weaned beef calves entering the feedlot environment the fecal prevalence of O157:H7 was found to be at 5% while 54% of tested hides were positive for O157:H7 (Arthur et al., 2009). However, a study investigating total STEC prevalence found 100% of 62 white veal calves were positive by ELISA for Shiga toxin 1 and/or Shiga toxin 2 (*stx1/2*) (Cristancho et al., 2008). Although isolation and genetic characterization of the *stx1/2* strains was not conducted for these samples, it does suggest that veal calves have the potential to harbor EHEC amongst the total STEC and could lead to hide contamination prevalence greater than that of O157:H7. The limited studies involving non-O157:H7 have identified EHEC of the serogroups O26, O103, O111, O118, and O145 as being associated with calves (Wieler et al., 1998; Pearce et al., 2004; Wang et al., 2014). This is likely not an exhaustive list of EHEC serogroups and additional studies are required to elucidate other EHEC serogroups found in veal calves. In addition, the current method for enumerating EHEC from veal calf samples uses direct plate counts on selective media and is limited to O157:H7, hence molecular assays to detect and enumerate EHEC associated with veal calves are required (Wang et al., 2014).

Culture based enumeration strategies, such as most probable number (MPN) or direct plate counts, can be a subjective and time-consuming process. Moreover, these assays could be impacted by EHEC that are viable but not culturable. Although, the contribution of viable but not culturable, EHEC to human disease is not fully known (Ramamurthy et al., 2014), molecular based assays would detect and include the unculturable EHEC in the enumeration. The use of real time PCR (qPCR) based enumeration methods are common for samples recovered from cattle. These assays primarily target a combination of the genes *stx1/2*, intimin (*eae*), *uidA, rfbE*, and *fliC* alleles for the detection and/or enumeration of O157:H7 and select Top 6 serogroups (Jacob et al., 2012; Wasilenko et al., 2012). However, these genes can be found separately in cells that are non-EHEC. Recently we used the *E. coli* attaching and effacing gene-positive conserved fragment 1 (*ecf1*), which is solely associated with EHEC (Boerlin et al., 1999; Becker and Groschel, 2014), as a gene target for the detection and enumeration of total EHEC directly from cattle feces using qPCR and reported a reliable limit of quantification of 1.25 × 10^3^ CFUs/mL (Luedtke et al., 2014). To provide a lower limit of detection, the *ecf1* target could be utilized in a molecular based modified MPN assay (Russo et al., 2014). In addition, the third generation of PCR termed digital PCR (dPCR) may offer an advantage to qPCR for the enumeration of total EHEC. In dPCR, Poisson based statistics are used to quantify absolute amounts of target DNA from tens of thousands of sub-nanoliter sized endpoint PCR reactions per sample. This reaction partitioning limits the interference of PCR inhibitors, allows for the detection of rare targets, and is not prone to amplification variability like replicate qPCR Cq values in the 35–40 range (Huggett et al., 2013; Marx, 2014).

Here we are the first to utilize three distinct molecular based assays detecting *ecf1* to enumerate total EHEC associated with veal hides at the abattoir and the resulting carcass. For the enumeration of total EHEC, identical pre-enrichment samples were run in parallel using our previously mentioned qPCR assay, a molecular based MPN assay, and by dPCR. Each assay had comparative strengths and weaknesses. The MPN assay provided the highest detection and enumeration rate while the qPCR assay allowed for the greatest dynamic range. Moreover, this is the first reported use of dPCR for the direct enumeration of bacteria from cattle samples. The application of a rapid and accurate assay for the enumeration of total EHEC associated with veal could provide a valuable tool, which is currently lacking.

## Materials and methods

### Sample collection

Paired hide and carcass samples were collected from 95 20- to 22-week-old formula fed veal calves at a veal processing plant in December 2013. Prior to any form of microbial intervention, hide samples were obtained after stunning using Speci-Sponges (Nasco, Fort Atkinson, WI), moistened with buffered peptone water (BPW) (BD, Sparks, MD), to swab an approximate 500 cm^2^ area over the breast-plate region. The sponges were passed (back and forth counting as one pass) five times either vertically or horizontally within the sample area and the sponge was flipped and passed five times in the remaining direction. The sponges were placed in respective Whirl-Pak bags (Nasco) containing 20 mL BPW. After the hide microbial intervention and removal, the respective carcass samples were obtained before any additional intervention in a similar fashion as the hide samples. An approximate 6000 cm^2^ area from the inside and outside round and the navel-plate-brisket-foreshank areas was swabbed and placed in Whirl-Pak bags with 20 mL BPW. All samples were secured in insulated coolers with ice packs for transport to the United States Meat Animal Research Center. All samples were processed the following day. Before removing aliquots for analysis, bacteria were dislodged from the sponges and suspended by toughly hand massaging the Whirl-Pak bags.

### qPCR

Prior to the enrichment of the hide and carcass samples, 20 μl of the respective sample was added to 180 μl of the BAX® system lysis buffer containing BAX® system protease and then prepared using the manufacturer's guidelines (DuPont, Wilmington, DE). All DNA samples were stored at −20°C prior to processing. A standard curve was generated using the *E. coli* O157:H7 reference strain EDL 932 (ATCC 43894) and divided into single use aliquots that were stored at −20°C. The EDL 932 standards, hide and carcass samples, and no template controls were run in duplicate reactions using the duplex qPCR assay targeting *eae* and *ecf1* as previously described (Luedtke et al., 2014). To normalize the quantification of the total EHEC across separate reaction plates, a pooled approach was used to develop the standard curve for enumeration. The pooled approach was reported to reduce uncertainty in the concentration of an unknown sample compared to a standard curve generated from a single instrument run since a similar mean is established from all of the instrument runs in the study analysis (Sivaganesan et al., 2010). The total EHEC was recorded as CFUs/mL and then converted to CFUs/100 cm^2^ using the previously described equation (Bohaychuk et al., 2011). In addition, the theoretical reliable limit of enumeration was calculated as CFUs/100 cm^2^ of swabbed hide and carcass using the previously described limit of enumeration of 1250 CFUs/mL for the *ecf1* target (Luedtke et al., 2014).

### Modified most probable number

A modified most probable number assay (MPN) was developed to increase the sensitivity of detection for the enumeration of total EHEC. From the Whirl-Pak bags, a 1 mL aliquot was transferred to 3 mLs of Tryptic Soy broth (TSB). For the hide samples, this initial dilution was used to create additional triplicate dilutions of 1:44, 1:484, and 1:5324 in BPW and incubated for 6 h at 42°C. Since the carcass samples likely had a lower starting amount of total EHEC, triplicate dilutions of 1:4, 1:44, and 1:484 were created and incubated as previously described. A 1 mL portion of each dilution for the hide and carcass samples was inoculated into a Roka G2 Sample Transfer Tube (Roka Bioscience, San Diego, CA). Samples were shipped on ice to the Roka Bioscience laboratory for analysis using the automated Atlas® system (Roka Bioscience), which targets *ecf1* mRNA for subsequent transcription mediated amplification and a hybridization protection assay. The MPN was determined from the number *ecf1* of positive replicates for each dilution and calculated using a freeware MPN calculator (Jarvis et al., 2010). The total EHEC was recorded as CFUs/mL and then converted to CFUs/100 cm^2^ using the previously described equation (Bohaychuk et al., 2011). Four carcass samples were incorrectly loaded and were removed from the sample set and comparative analysis.

### dPCR

Since this is the first description of using dPCR to enumerate bacteria directly from environmental sources, select samples with enumerable total EHEC from the MPN and qPCR were utilized for absolute enumeration. The absolute enumeration of total EHEC from 26 hide and 16 carcass pre-enrichment samples was performed in 15 μl reactions. The reactions contained 7.5 μl of the Quantstudio™ 3D Digital PCR Master Mix (Applied Biosystems® by Life Technologies, Carlsbad, CA), 2 μl of DNA, 3.7 μl of PCR grade H_2_O, and the addition of the *eae* and *ecf1* primers and probes at the previously described concentrations (Luedtke et al., 2014). A 14.5 μl aliquot of each reaction was loaded onto respective QuantStudio™ 3D Digital PCR 20K Chips (Applied Biosystems® by Life Technologies), which has 20,000 wells that can accommodate a 865 pL reaction per well, using the automated QuantStudio™ 3D Digital PCR Chip Loader (Applied Biosystems® by Life Technologies). No template controls and a field sample that screened negative by qPCR for both targets were also included for each run. All loaded chips were assembled according to the manufacturer's recommendations (Applied Biosystems® by Life Technologies). For the amplification of the DNA targets, the loaded chips were placed on a flat block Gene Amp® 9700 thermocycler (Applied Biosystems® by Life Technologies) and used the cycling conditions 96°C for 10 min, with 39 cycles of 59°C for 2 min and 98°C for 30 s, a hold at 59°C for 2 min, and a 4°C hold. After the thermocycling was completed, the chips were allowed to warm to room temperature and analyzed within 1 hr after removal from the thermocylcer using the QuantStudio™ 3D Digital PCR Instrument (Applied Biosystems® by Life Technologies). Samples where the chip leaked the QuantStudio™ 12K Flex OpenArray® Immersion Fluid (Applied Biosystems® by Life Technologies) during thermocycling were redone using a new chip. Further analysis of the data was performed using the Quantstudio™ 3D AnalysisSuite™ version 2.0.0 (Applied Biosystems® by Life Technologies). All chips were analyzed for the quality of the read and adjusted to a quality threshold of 0.6 for an increased stringency. The fluorescent threshold was adjusted according to the no template control and the sample that screened negative for both targets by qPCR. These controls served as a baseline for background fluorescence of the FAM and MAXN dyes. The fluorescent threshold was adjusted above the background and then universally for *eae* and *ecf1* across all samples due to the mono-modal peak associated with samples that contain a limited amount of target DNA. The total EHEC was recorded as CFUs/mL and then converted to CFUs/100 cm^2^ using the previously described equation (Bohaychuk et al., 2011).

### Sample enrichment

To determine the prevalence of total EHEC in the hide and carcass samples, the Whirl-Pak bags were supplemented with 80 mL of TSB and incubated for 6 h at 42°C. The enriched samples were processed for qPCR as previously described and a multiplex qPCR assay was used to detect the presence of *eae, ecf1*, and *stx1/2* (Luedtke et al., 2014).

### Statistics

GraphPad Prism 6 (GraphPad Software, La Jolla, CA) was used to determine the 95% confidence intervals for the qPCR assays, construct the Bland-Altman plots, Pearson correlation coefficients, and was used to calculate significant differences using the χ^2^ test. *P*-values < 0.05 were considered significant.

## Results

### qPCR standard curve and enumeration of total EHEC from veal hide and carcass samples

**Figure 1:**
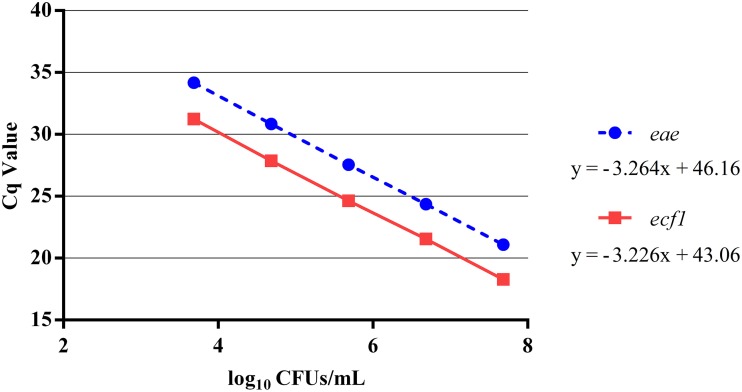
**Five log standard curve for ***eae*** and ***ecf1*** that was generated from the average of the pooled Cq values**. Individual aliquots of the same five log standard curve of the EDL 932 gDNA were loaded in duplicate for each plate during the enumeration of the total EHEC concentration in the paired veal hide and carcass samples. The Cq values for *eae* and *ecf1* at each dilution were pooled and averaged and plotted against the representative EDL 932 concentration. Error bars indicate the average standard deviation.

Using a pooled approach to develop the qPCR standard curve for enumerating total EHEC provided a reproducible curve over the five log dilution series (Table 1). Overall, the PCR efficiency for *eae* and *ecf1* was 102 and 104%, respectively (Figure 1). In addition, the no template controls were consistently negative across all qPCR assays.

**Table 1:** **Average Cq values and coefficients of variability from pooled standard curves collected during the enumeration of total EHEC from veal hides and carcasses**.

**log_10_ CFUs/mL**	***eae***		***ecf1***	
	**Cq ± SD^a^**	**CV^b^**	**Cq ± SD**	**CV**
7.69	21.09 ± 0.09	5.66%	18.27 ± 0.04	2.59%
6.69	24.37 ± 0.11	6.99%	21.55 ± 0.10	6.49%
5.69	27.54 ± 0.04	2.78%	24.64 ± 0.05	3.24%
4.69	30.83 ± 0.12	7.28%	27.87 ± 0.11	6.95%
3.69	34.18 ± 0.41	25.21%	31.24 ± 0.50	30.85%

a *The average Cq value ± the standard deviation*.

b CV, coefficient of variability

Total EHEC was enumerable in 30 (32%) of the 95 pre-enrichment hide samples using the duplex qPCR assay. Based on the *ecf1* target, the amount of total EHEC ranged from approximately 34 to 91,412 CFUs/100 cm^2^ (95% CI 4–113,460 CFUs/100 cm^2^). However, 26 (87%) of the enumerable samples were below the calculated reliable limit of enumeration of 5000 CFUs/100 cm^2^ for the hide samples. Despite being below the reliable limit of enumeration, total EHEC could be enumerated based on extrapolation from the standard curve. However, some samples had a single Cq value from the duplicate reactions. This also occurred amongst the carcass samples. From the 95 pre-enrichment carcass samples, total EHEC was enumerable in 16 (17%) samples and all of the enumerable samples had a concentration below the calculated reliable limit of quantification of approximately 417 CFUs/100 cm^2^ for the carcass samples. Using the *ecf1* target, the amount of total EHEC in the carcass samples ranged from approximately 4–275 CFUs/100 cm^2^ (95% CI 3–953 CFUs/100 cm^2^).

### MPN enumeration of total EHEC from veal hide and carcass samples

The MPN assay was able to enumerate total EHEC in 46 (48%) of the 95 hide swab samples, and indicated a concentration of total EHEC ranging from approximately 1 to greater than 17,022 CFUs/100 cm^2^ (95% CI 0.4–72,000 CFUs/100 cm^2^). For the MPN analysis of total EHEC on the carcasses, 91 samples were included. As observed with the qPCR assay, the MPN assay indicated that the carcass samples have a low concentration of total EHEC, which ranged from approximately 0.1–1 CFU/100 cm^2^ (95% CI 0.02–4 CFUs/100 cm^2^) from 26 (29%) of the samples.

### Comparison of the qPCR and MPN for the enumeration of total EHEC from veal hide and carcass samples

**Figure 2:**
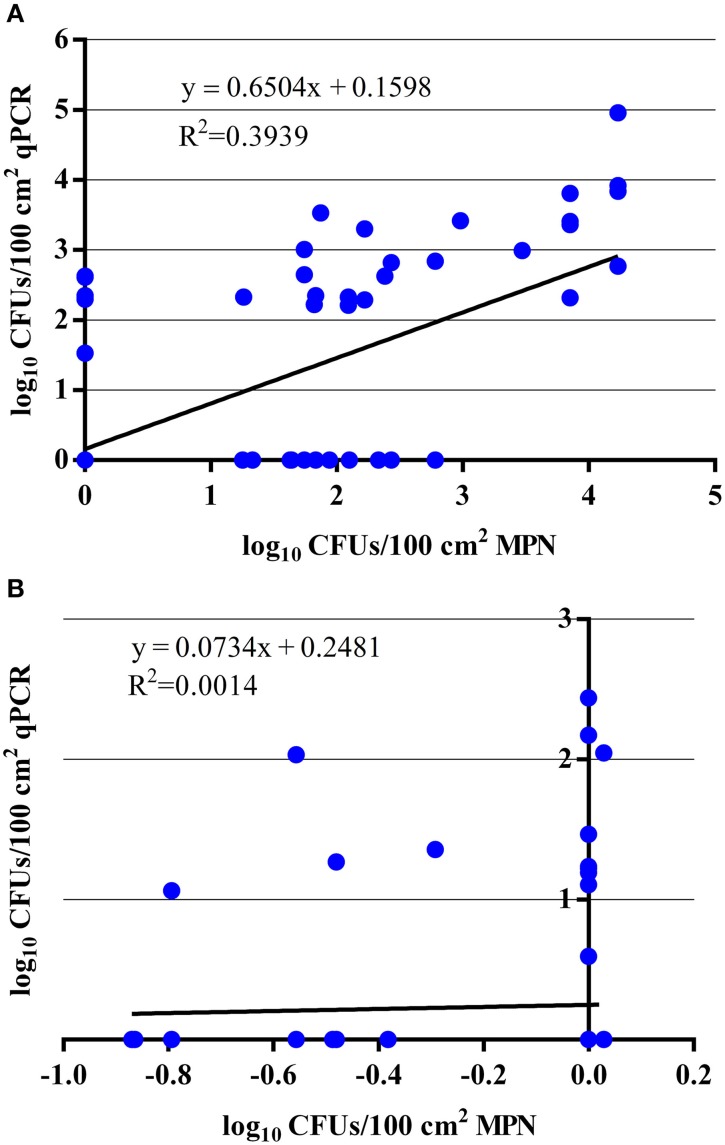
**Comparison of qPCR and MPN assays for the enumeration of total EHEC from paired veal hide and carcass samples. (A)** Regression analysis comparing total EHEC concentrations from 95 hide samples that were enumerated by the qPCR and MPN assays targeting *ecf1*. **(B)** Regression analysis comparing total EHEC concentrations from 91 carcass samples that were enumerated by the qPCR and MPN assays targeting *ecf1*.

**Figure 3:**
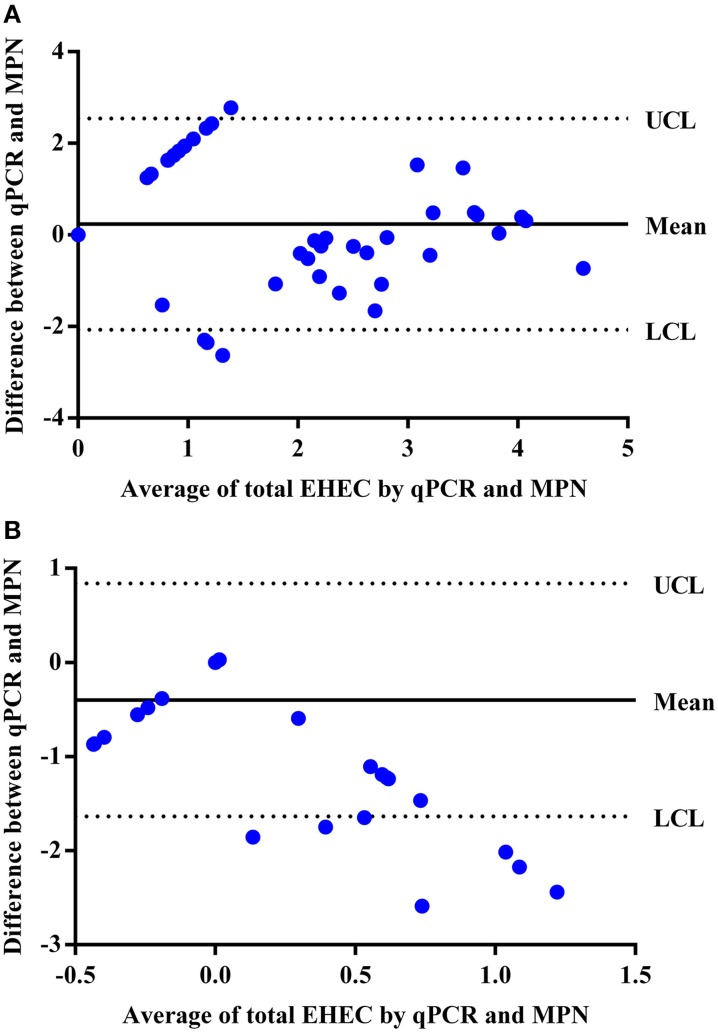
**Bland-Altman plot to determine if the qPCR and MPN assays are interchangeable for the enumeration of total EHEC from veal hide and carcass samples. (A)** Hide samples were enumerated by the qPCR and MPN assays and compared for agreeability between the two methods based on the average total EHEC enumerated (log_10_ CFUs/100 cm^2^) for a respective sample using the qPCR and MPN assays and the difference in enumeration values (log_10_ CFUs/100 cm^2^) between the assays. **(B)** Carcass samples were enumerated by the qPCR and MPN assays and compared for agreeability between the two methods based on the average total EHEC enumerated (log_10_ CFUs/100 cm^2^) for a respective sample using the qPCR and MPN assays and the difference in enumeration values (log_10_ CFUs/100 cm^2^) between the assays. UCL and LCL indicate the 95% upper confidence level and 95% lower confidence level, respectively. The mean is the average difference between the two methods.

By comparing the qPCR assay to the MPN assay, the qPCR assay was able to enumerate total EHEC in 23 (50%) of the hide samples that were also enumerable with the MPN assay. In addition, the qPCR assay was able to enumerate total EHEC in 7 (7%) of the hide samples that was not enumerable by the MPN assay. Samples with an enumerable amount of total EHEC were within the same log_10_ value for 10 (43%) samples while the remaining 13 (57%) samples were within approximately one to two orders of magnitude difference between the qPCR and MPN assays (Table 2). Samples only enumerable by either qPCR or the MPN assay were below 3 log_10_ CFUs/100 cm^2^. Regression analysis between the qPCR and MPN assays for the hide samples was significant (*p* < 0.00001) with a Pearson correlation coefficient of 0.63 (Figure 2A) and the Bland-Altman plot indicates that 92 (97%) of the hide samples were within the 95% confidence interval. This suggests that the two methods are interchangeable for the enumeration of total EHEC from hide samples (Figure 3A). Amongst the carcass samples, the qPCR assay was able to enumerate total EHEC in 10 (11%) samples not enumerable by the MPN assay, while the MPN assay was able to enumerate total EHEC in 21 (23%) samples not enumerable by the qPCR assay. In addition, 5 (5%) samples had a concentration of total EHEC that was enumerable by both assays. The Pearson correlation coefficient for the qPCR and MPN assays on the carcass samples was 0.04, which indicates that a relationship between the methods does not exist (Figure 2B). The Bland-Altman plot supports that the two methods are not interchangeable since less than 95% of the samples were within the confidence interval (Figure 3B). The differences in the ability to enumerate total EHEC between the qPCR and MPN assays could be explained by the methodology and limitations of each assay.

**Table 2:** **Distribution of total EHEC enumerated from paired veal hides and carcasses using qPCR and MPN assays**.

**log_10_ CFUs/100cm^2^**	**Hides**		**log_10_ CFUs/100cm^2^**	**Carcasses**	
	**qPCR (%)**	**MPN (%)**		**qPCR (%)**	**MPN^a^ (%)**
4	1 (1)	4 (4)	2	4 (4)	0 (0)
3	9 (9)	5 (5)	1	11 (12)	0 (0)
2	19 (20)	15 (16)	< 1	1 (1)	26 (29)
1	1 (1)	22 (23)	NE^b^	79 (83)	65 (71)
NE^b^	65 (69)	49 (52)			

a *n = 91*.

b *NE, No enumeration*.

### dPCR analysis of select veal hide and carcass samples for the enumeration of total EHEC

**Figure 4:**
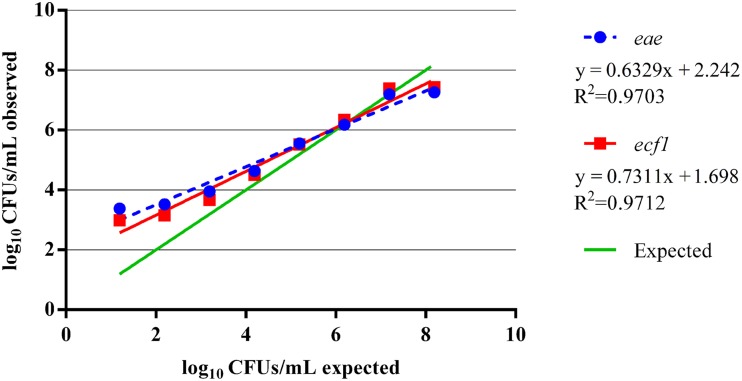
**Performance of the dPCR assays over an eight log standard curve**. A separate eight log standard curve was created using gDNA from the EDL 932 reference strain. The standard curve was run on a single dPCR chip and targeted *eae* and *ecf1* and plotted against the expected input target concentration.

Additional analysis of the total EHEC enumeration observations was performed on select hide and carcass samples using dPCR. To determine the capabilities of dPCR, a separate eight log standard curve from approximately 8.19–1.19 log_10_ CFUs/mL was created using the EDL 932 reference strain. From this standard curve, it was found, for both targets, that the dPCR assay was within the same log value as the expected inoculums for dilutions containing approximately 3–7 log_10_ CFUs/mL (Table 3). At the dilution with an expected 8.19 log_10_ CFUs/mL, the dPCR assay indicated approximately 7.26 log_10_ CFUs/mL based on the *eae* target while the *ecf1* target indicated 7.44 log_10_ CFUs/mL. Thus, the upper limit of this dPCR assay is approximately within the 7 log_10_ CFUs/mL range. Moreover, the lower limit was found to be approximately 3 log_10_ CFUs/mL since at the expected dilution of 2.19 log_10_ CFUs/mL the concentration of the *eae* and *ecf1* targets was 3.52 log_10_ and 3.15 log_10_ CFUs/mL, respectively (Figure 4). Moreover, at the expected dilutions of 2.19 and 1.19 log_10_ CFUs/mL, the precision for both targets was above 100% (Table 3). Analyzing the same standard curve template DNA by qPCR showed a similar trend, from dilutions containing approximately 7.19 to 3.19 log_10_ CFUs/mL, as the dPCR. The respective efficiency for *eae* and *ecf1* over the five log curve was 92% and 94%, with an R^2^ of 0.999 for both targets (Supplementary Figure 1).

**Table 3:** **Expected reference strain input and returned output for concentration of ***eae*** and ***ecf1*** targets from dPCR assay**.

**Input log_10_ CFUs/mL**	***eae***		***ecf1***	
	**Output log_10_ CFUs/mL**	**dPCR precision (%)**	**Output log_10_ CFUs/mL**	**dPCR precision (%)**
8.19	7.26	1.84	7.44	2.18
7.19	7.20	1.80	7.39	2.02
6.19	6.18	3.70	6.35	3.11
5.19	5.55	7.50	5.52	7.71
4.19	4.64	22.40	4.50	26.83
3.19	3.95	56.78	3.67	85.86
2.19	3.52	109.76	3.15	210.06
1.19	3.38	140.26	2.99	299.85

**Figure 5:**
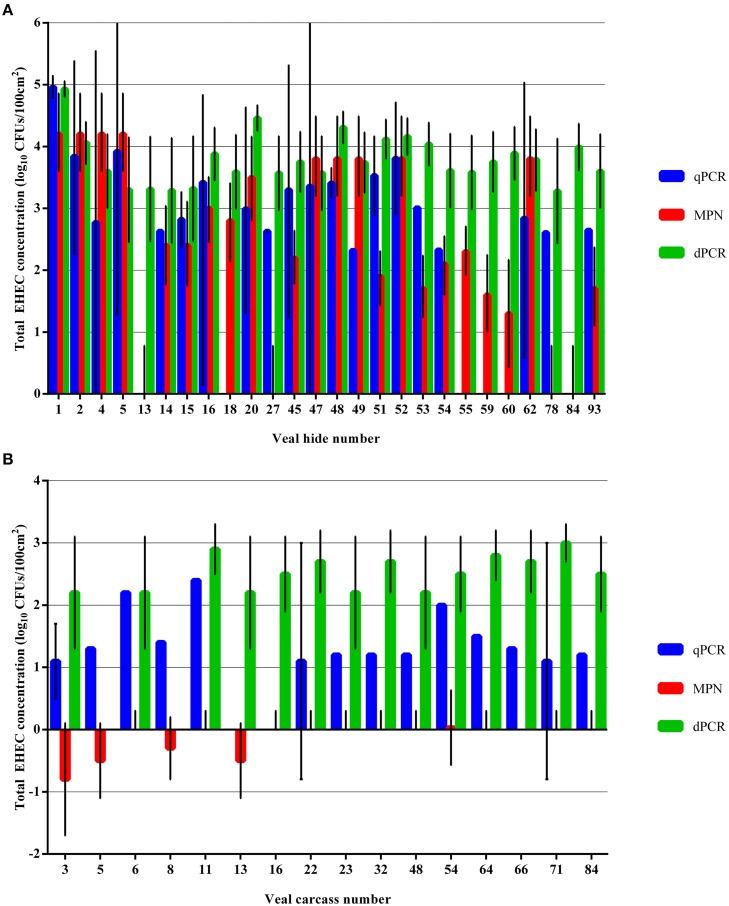
**Comparison of total EHEC estimated from select veal hide and carcass samples using qPCR, MPN, and dPCR assays targeting ***ecf1***. (A)** Total EHEC from selected hide samples were enumerated using the dPCR assay and compared to the enumeration values returned by the qPCR and MPN assays. **(B)** Total EHEC from selected carcass samples were enumerated using the dPCR assay and compared to the enumeration values returned by the qPCR and MPN assays. Error bars indicate the 95% confidence interval for each sample. A 95% confidence interval was not calculated for samples with a single Cq value from the qPCR assay.

The dPCR assay tended to overestimate the concentration of total EHEC in the select hide and carcass samples that were indicated previously by the qPCR and MPN assays to have less than 3 log_10_ CFUs/100 cm^2^ (Figures 5A,B). However, for the hide samples with total EHEC over 3 log_10_ CFUs/100 cm^2^ the dPCR assay was in the same order of magnitude for 5 (50%) and 6 (60%) of the samples enumerated by either qPCR or the MPN assay, respectively. In three samples, 4, 49, and 62, the dPCR assay estimated the total EHEC concentration closer to the concentration enumerated by the MPN assay while the qPCR assay determined the EHEC concentration to be greater than approximately one magnitude lower (Supplementary Table 1). In addition, the samples 45, 51, and 53 were closer in the estimated concentration between the qPCR and dPCR assay than the MPN assay (Supplementary Table 1). The carcass samples selected for dPCR analysis all had total EHEC below 3 log_10_ CFUs/100 cm^2^ as determined by the qPCR and MPN assays. Despite the low level of total EHEC, the dPCR assay determined the concentration within the same log_10_ for approximately 3 (19%) and was an order of magnitude higher or lower for 10 (62%) and two orders higher for 3 (19%) of the samples as the qPCR assay (Supplementary Table 2). The MPN assay estimated the total EHEC concentration on the carcasses at two to three orders of magnitude lower than the qPCR and dPCR assays (Supplementary Table 2).

### Prevalence of total EHEC in veal hide and carcass enrichments

To determine the prevalence of total EHEC in the hide and carcass samples, the samples were enriched and a multiplex qPCR assay targeting *eae, ecf1*, and *stx1/2* was performed. From the hide samples, total EHEC, which are positive for all three targets, was detected in 72 (76%) of the samples while 5 (5%) samples were positive for only *eae* and *ecf1*. In addition, *eae* and *stx1/2* was detected in 12 (13%) of the samples while *eae* alone was detected in 6 (6%) samples. Using a χ^2^ test, significantly (*p* = < 0.05) more carcass samples were positive for all three targets than the hide samples with 86 samples. This accounted for approximately 91% of the carcass samples while 3 (3%) samples had detectable amounts of only *eae* and *ecf1*. Four (4%) of the carcass samples had detectable amounts of only *eae* and *stx1/2* and 1 (1%) sample contained only *eae* while 1 (1%) sample was negative for all three targets. The average post enrichment Cq values for the hide and carcass samples that were positive for all three targets were compared. The hide samples had an average Cq value ± the standard deviation for *eae, ecf1*, and *stx1/2* of 27.4±1.6, 30.8±2.7, and 29.0±3.0 respectively. For the carcass samples, the respective average Cq values were 33.4±2.9, 34.7±1.9, and 34.2±3.1 for *eae, ecf1*, and *stx1/2*. A correlation between the pre-enrichment enumeration values and the post-enrichment Cq values for hide and carcass samples was not identified (data not shown).

## Discussion

Pathogenic *E. coli* remains a constant concern for food safety and human health with an emphasis on the most severe pathotype, EHEC (Palaniappan et al., 2006). A systematic review and meta-analysis covering 62 years of published reports indicates a stable and continued association of EHEC with calves (Kolenda et al., 2015), yet veal calves have received limited study toward EHEC detection and enumeration methods (Wang et al., 2014). To address these issues, we sought to investigate the use of qPCR, molecular MPN, and dPCR assays to detect and enumerate total EHEC from paired pre-intervention veal calf hides and carcasses.

Studies utilizing qPCR to detect and enumerate EHEC primarily focus on a single EHEC such as O157:H7 or target virulence genes that can be independently possessed by non-EHEC in a polymicrobial matrix. This use of potential non-conjoined targets results in false positives and an over estimation of the true EHEC population (Jacob et al., 2012). Recently, Livezey et al. (2015) reported the use of *ecf1* as a target for the detection of total EHEC in beef samples. That study detailed the specificity of *ecf1* in *E*. *coli* possessing *eae, stx1/2*, and *ehxA*, which is applicable to determining the total EHEC load in a sample. In addition, qPCR has primarily been used to detect and enumerate specific EHEC from cattle feces, while the application of qPCR on direct hide and carcass samples is unreported. This is likely due to the low concentration of a specific EHEC serogroup within a defined area on the hide or carcass (Arthur et al., 2007), and the intrinsic limit of detection and enumeration for qPCR, which can range between 10^3^ and 10^4^ CFUs/mL. Indeed, based on the surface area sampled in this study, to reach the theoretical limit of detection and enumeration comparable to the previously determined reliable limit of 1250 CFUs/mL (Luedtke et al., 2014) a concentration of 5000 and 417 CFUs/100 cm^2^ is required for the hide and carcass samples, respectively.

While we could not find any published reports of using qPCR to determine the total EHEC concentration on cattle or veal hides or carcasses, a PCR-MPN based investigation of potential total EHEC from 11 head of cattle found the respective average concentration on hides and carcasses to be approximately 15662 and 123 CFUs/100 cm^2^ when using the average of the targets *eae* and *ehxA* for the enumeration regardless of the cattle diet (Gilbert et al., 2008). Analyzing the data in this manner provides a better normalization to our enumeration targeting *ecf1* as *ecf1* is in a 1:1 relationship with *E. coli* possessing both *eae* and *ehxA* (Livezey et al., 2015). Using this methodology suggests that our use of qPCR to enumerate total EHEC from veal hides and carcasses may under estimate the total EHEC load (Gilbert et al., 2008). Moreover, determining the precise amount of total EHEC in low concentration samples is difficult due to the Monte Carlo effect (Bustin and Nolan, 2004) as respectively 87 and 100% of the enumerable hide and carcass samples were below the reliable limit of enumeration and some samples returned a single Cq value for the duplicate reactions.

Variations of the MPN assay have been described. These modifications incorporate a combination of immunomagnetic separation and PCR to increase sensitivity. However, these MPN assays primarily focus on the enumeration of O157 and O26 from feces (Widiasih et al., 2004; Stephens et al., 2007; Guy et al., 2014) while two studies have investigated the total potential EHEC in feces and on hides and carcasses (Gilbert et al., 2005, 2008). With our dilution range for the hide and carcass samples the enumeration of total EHEC was mostly one to two logs below the estimates of average potential EHEC reported by Gilbert et al. (2008). However, Gilbert et al. (2008) was analyzing cattle and differences in cattle versus veal EHEC hide carriage and carcass processing techniques may exist in addition to physiological and environmental differences (Cristancho et al., 2008; Wang et al., 2014). In addition, the use of an *a priori* dilution scheme, like used here, for the MPN assays highlights the limitations in the ability to enumerate total EHEC at the upper and lower concentrations from a diversity of samples. To encompass a diverse sample set by expanding the MPN dilution range would reduce the throughput and increase the cost per sample of large analyses.

Comparatively, the MPN assay was able to detect and enumerate total EHEC from 17 (17%) more hide and 11 (12%) more carcass samples than the qPCR assay. However, the qPCR assay was able to enumerate total EHEC in 7 hide and 11 carcass samples that were not enumerable by the MPN assay. The additional samples enumerable by PCR could be due to differences in the detection methods. In the qPCR assay, all amplifiable DNA contributes to the enumeration, which would include free DNA and DNA from injured *E. coli*, while the MPN assay targets *ecf1* mRNA transcripts and would likely only enumerate viable cells. In addition, the targeting of mRNA would improve the sensitivity of the assay since more template would be available for detection, which is likely why more hide and carcass samples were enumerable than with the qPCR assay. Despite the detection and enumeration advantage of the MPN assay, the indication, from the Bland Altman plot, that the MPN and qPCR assay are interchangeable for hide enumerations would save a considerable amount of time and resources when using the qPCR assay. The reliable detection and enumeration of total EHEC from carcasses offers an additional challenge due to the inherently low concentrations of total EHEC that resulted in a difference of 2 to 3 orders of magnitude between the qPCR and MPN assays.

To overcome the low concentrations of total EHEC, we sought to investigate dPCR. Digital PCR has been previously shown to be insensitive to PCR inhibitors and perform similarly to qPCR on environmental samples, but does not require a standard curve to enumerate the target DNA concentration (Blaya et al., 2015; Kinz et al., 2015). Our analysis of a dilution curve using dPCR showed a similarity to qPCR in the enumeration of the gene targets with the expected input of approximately 10^7^ and 10^3^ CFUs/mL being within the same order of magnitude for the two methods. However, based on precision, the dynamic range of the dPCR was limited compared to the qPCR assay. To increase the dynamic range toward lower target concentrations, additional dPCR replicates are required to lower the percent precision within an acceptable range (Blaya et al., 2015; Majumdar et al., 2015). The requirement to perform additional dPCR reactions for a single sample with a high percent precision value could be cost inhibitory for commercial application; hence additional dPCR reactions were not performed in this study.

False positive and negative reactions at lower target concentrations can impact the accurate enumeration of the target (Majumdar et al., 2015) as can incidental that pipetting errors between dilutions that change the expected concentration, which the absolute enumeration of dPCR would detect and provide a true estimation (Kishida et al., 2014). dPCR is less prone to error due to stochastic effects like qPCR at low target concentrations. This was evident in hide samples 4, 49, and 62 as the qPCR assay had a high standard deviation between duplicates while the dPCR was in agreement with the concentration estimated by the MPN assay. To our knowledge, this is the first report of using dPCR to enumerate total EHEC from an environmental source and without using previous DNA purification methods.

Our analysis of the 95 hide samples after enrichment indicted total EHEC prevalence at 76%, which is below the 94% average prevalence of the Top 6 EHEC on 132 veal hides as reported by Wang et al. (2013). This difference in prevalence could be due to the origin of the calves and/or the detection method utilized. The method used by Wang et al. (2013) identifies *stx1/2* and single-nucleotide polymorphisms associated with the Top 6 serogroup and *eae*. However, the analysis of results from a polymicrobial sample like hides could result in false positives as we observed 95 (100%) of the hide samples possessing *eae* while 18 (19%) lacked *ecf1* and 12 (67%) of these samples also contained *stx1/2*. This would cause a misinterpretation of true positives if all targets were possessed by separate bacteria, and has been indicated to occur, using the same method, during the detection of the Top 7 on beef hides (Stromberg et al., 2015). Interestingly, the carcass samples had significantly more (*P* < 0.05) samples possessing all three gene targets. With these total EHEC detections coming from an enrichment of the sample, it suggests that the hide intervention utilized maybe not be effective and/or the equipment and procedures utilized for hide removal facilitate further total EHEC transmission (United States Department of Agriculture, and Food Safety and Inspection Service, 2013). However, the comparison of Cq values, although not a fully quantitative method, between the hide and carcass sample sets does provide a generalized estimation of the total EHEC population prior to enrichment with regards to the background microflora population and enrichment media (Vimont et al., 2007). With this methodology, the pre-enrichment hide samples likely started with a total EHEC near the detection/enumeration limit while the carcass samples were below the limit (Luedtke et al., 2014) as we observed for the qPCR, MPN, and dPCR assays. Moreover, differences in the background microflora between samples would explain why a correlation between the enumeration value and the Cq value was not identified (Vimont et al., 2007). In addition, samples possessing *eae* and *ecf1*, which are classified as atypical EPEC (Livezey et al., 2015), remain a concern due to the potential for these cells to regain *stx1/2* at later point (Bielaszewska et al., 2007) but would not be identified using the conventional detection and enumeration methods.

In conclusion, veal has received limited research pertaining to food safety despite being a significant source of total EHEC. This study is the first to enumerate total EHEC from paired pre-intervention veal hides and carcasses. Each of the methods utilized for detection and enumeration had benefits and drawbacks. The qPCR assay was easy to use and offered a high throughput, but the inherent low concentration of total EHEC on the hides and carcasses limited the accuracy and the requirement of a standard curve limits consistency and number of samples loaded. Our MPN assay allowed for the detection of viable cells and offers a lower limit of detection; however, this sample size inhibited the throughput and required a different range of dilutions. Digital PCR offers advantages of a medium throughput and no standard curve, although the dynamic range of dPCR is limited and to improve precision would require additional analysis of samples with a total EHEC concentration below the reliable limit. When attempting to enumerate low concentrations of total EHEC, the MPN assay would provide the most accurate results while the qPCR and dPCR assays would be effective in determining veal with hide concentrations above approximately 5000 CFUs/100 cm^2^ within 4 h. By rapidly determining highly contaminated calves, these animals could be restricted to end of the day production or receive an increased focus during intervention. Moreover, it was unexpected to detect a higher prevalence of total EHEC on the carcass samples, but the use of an additional carcass intervention may eliminate the risk imposed by the low concentration of total EHEC on the carcass. However, further research tracking the potential spread of total EHEC from a veal hide to the resulting carcasses in combination with our detection and enumeration assays would provide a better understanding of why and how veal trim has an higher association with total EHEC than beef trim.

## Author contributions

BL and JB contributed equally to the designing of experiments, conducting assays, data analysis, and drafting the manuscript.

### Conflict of interest statement

The authors declare that the research was conducted in the absence of any commercial or financial relationships that could be construed as a potential conflict of interest.
