# Supplementary material for: Comparison of methods for the enumeration of enterohemorrhagic Escherichia coli from veal hides and carcasses
Source: Front Microbiol. 2015 Sep 29;6:1062. doi: 10.3389/fmicb.2015.01062 (PMC4586433; doi:10.3389/fmicb.2015.01062)
Supplement: Supplementary file 1 [file Table1.DOCX]

***Supplementary Material***

**Comparison of methods for the enumeration of enterohemorrhagic *Escherichia coli* from veal hides and carcasses**

**Brandon E. Luedtke and Joseph M. Bosilevac^*^**

*** Correspondence:** Joseph M. Bosilevac, U. S. Department of Agriculture, Agricultural Research Service, Roman L. Hruska U. S. Meat Animal Research Center, State Spur 18D, Clay Center, Nebraska 68933-0166, USA, E‑mail: [mick.bosilevac@ars.usda.gov](mailto:mick.bosilevac@ars.usda.gov).

**Supplementary Table 1. Log transformed values of total EHEC/100 cm^2^ enumerated from select hide samples using MPN, qPCR, and dPCR assays^a^.**

| Sample | qPCR | | MPN | | dPCR | |
| --- | --- | --- | --- | --- | --- | --- |
|  | log_10_ CFUs/100cm^2^ | 95% CI | log_10_ CFUs/100cm^2^ | 95% CI | log_10_ CFUs/100cm^2^ | 95% CI |
| 1 | 4.9 | 4.8-5.1 | 4.2 | 3.6-4.9 | 4.9 | 4.8-5.1 |
| 2 | 3.8 | 2.3-5.4 | 4.2 | 3.6-4.9 | 4.1 | 3.7-4.4 |
| 4 | 2.8 | -4.5-9.5 | 4.2 | 3.6-4.9 | 3.6 | 2.9-4.2 |
| 5 | 3.9 | 1.3-6.5 | 4.2 | 3.6-4.9 | 3.3 | 2.5-4.2 |
| 13 | 0.0 | 0.0 | 0.0 | 1.7-3.0 | 3.3 | 2.5-4.2 |
| 14 | 2.6 | 0.0 | 2.4 | 0.0-0.8 | 3.3 | 2.4-4.1 |
| 15 | 2.8 | 2.4-3.3 | 2.4 | 1.7-3.1 | 3.3 | 2.5-4.2 |
| 16 | 3.4 | 0.1-4.8 | 3.0 | 2.5-3.5 | 3.9 | 3.5-4.3 |
| 18 | 0.0 | 0.0 | 2.8 | 2.1-3.4 | 3.6 | 2.9-4.2 |
| 20 | 3.0 | 1.3-4.6 | 3.5 | 2.8-4.2 | 4.5 | 4.3-4.7 |
| 27 | 2.6 | 2.6-2.6 | 0.0 | 0.0-0.8 | 3.6 | 2.9-4.2 |
| 45 | 3.3 | 1.2-5.3 | 2.2 | 1.8-2.6 | 3.8 | 3.3-4.2 |
| 47 | 3.4 | -0.3-6.9 | 3.8 | 3.2-4.5 | 3.5 | 2.9-4.2 |
| 48 | 3.4 | 3.2-3.7 | 3.8 | 3.2-4.5 | 4.3 | 4.1-4.6 |
| 49 | 2.3 | 0.0 | 3.8 | 3.2-4.5 | 3.7 | 3.2-4.2 |
| 51 | 3.5 | 2.9-4.2 | 1.9 | 1.4-2.3 | 4.1 | 3.8-4.4 |
| 52 | 3.8 | 2.9-4.7 | 3.8 | 3.2-4.5 | 4.2 | 3.9-4.5 |
| 53 | 3.0 | 0.0 | 1.7 | 1.2-2.2 | 4.0 | 3.7-4.4 |
| 54 | 2.3 | 0.0 | 2.1 | 1.6-2.6 | 3.6 | 3.0-4.2 |
| 55 | 0.0 | 0.0 | 2.3 | 1.9-2.7 | 3.6 | 2.9-4.2 |
| 59 | 0.0 | 0.0 | 1.6 | 1.0-2.3 | 3.7 | 3.3-4.2 |
| 60 | 0.0 | 0.0 | 1.3 | 0.4-2.1 | 3.9 | 3.5-4.3 |
| 62 | 2.8 | 0.6-5.0 | 2.8 | 2.1-3.4 | 3.8 | 3.3-4.3 |
| 78 | 2.6 | 0.0 | 0.0 | 0.0-0.8 | 3.3 | 2.4-4.1 |
| 84 | 0.0 | 0.0 | 0.0 | 0.0-0.8 | 3.9 | 3.6-4.4 |
| 93 | 2.7 | 0.0 | 1.7 | 1.1-2.4 | 3.6 | 3.0-4.2 |

^a^ All assays used the *ecf1* target for enumeration

^b^ qPCR data with a 0.0 95% CI indicates either 1 or 0 of the duplicates provided a Cq value
