# Supplementary material for: Comparison of methods for the enumeration of enterohemorrhagic Escherichia coli from veal hides and carcasses
Source: Front Microbiol. 2015 Sep 29;6:1062. doi: 10.3389/fmicb.2015.01062 (PMC4586433; doi:10.3389/fmicb.2015.01062)
Supplement: Supplementary file 2 [file Table2.DOCX]

***Supplementary Material***

**Comparison of methods for the enumeration of enterohemorrhagic *Escherichia coli* from veal hides and carcasses**

**Brandon E. Luedtke and Joseph M. Bosilevac^*^**

*** Correspondence:** Joseph M. Bosilevac, U. S. Department of Agriculture, Agricultural Research Service, Roman L. Hruska U. S. Meat Animal Research Center, State Spur 18D, Clay Center, Nebraska 68933-0166, USA, E‑mail: [mick.bosilevac@ars.usda.gov](mailto:mick.bosilevac@ars.usda.gov).

**Supplementary Table 2. Log transformed values of total EHEC/100 cm^2^ enumerated from select carcass samples using MPN, qPCR, and dPCR assays^a^**

| Sample | qPCR | | MPN | | dPCR | |
| --- | --- | --- | --- | --- | --- | --- |
|  | log_10_ CFUs/100cm^2^ | 95% CI | log_10_ CFUs/100cm^2^ | 95% CI | log_10_ CFUs/100cm^2^ | 95% CI |
| 3 | 1.1 | 0.4-1.7 | -0.8 | -1.7-0.1 | 2.2 | 1.3-3.0 |
| 5 | 1.3 | 0.0 | -0.5 | -1.1-0.1 | 0.0 | 0.0 |
| 6 | 2.2 | 0.0 | 0.0 | 0-0.2 | 2.2 | 1.4-3.1 |
| 8 | 1.4 | 0.0 | -0.3 | -0.8-0.2 | 0.0 | 0.0 |
| 11 | 2.4 | 0.0 | 0.0 | 0-0.2 | 2.9 | 2.5-3.3 |
| 13 | 0.0 | 0.0 | -0.5 | -1.1-0.1 | 2.2 | 1.4-3.1 |
| 16 | 0.0 | 0.0 | 0.0 | 0-0.2 | 2.5 | 1.9-3.1 |
| 22 | 1.1 | -0.8-3.0 | 0.0 | 0-0.2 | 2.7 | 2.2-3.2 |
| 23 | 1.2 | 0.0 | 0.0 | 0-0.2 | 2.2 | 1.4-3.1 |
| 32 | 1.2 | 0.0 | 0.0 | 0-0.2 | 2.7 | 2.2-3.2 |
| 48 | 1.2 | 1.2-1.2 | 0.0 | 0-0.2 | 2.2 | 1.3-3.1 |
| 54 | 2.0 | 0.0 | 0.03 | -0.6-0.7 | 2.5 | 1.9-3.1 |
| 64 | 1.5 | 0.0 | 0.0 | 0-0.2 | 2.8 | 2.4-3.2 |
| 66^c^ | 1.3 | 0.0 | ND | ND | 2.7 | 2.2-3.2 |
| 71 | 1.1 | -0.8-3.0 | 0.0 | 0-0.2 | 3.0 | 2.7-3.4 |
| 84 | 1.2 | 0.0 | 0.0 | 0-0.2 | 2.5 | 1.9-3.1 |

^a^ All assays used the *ecf1* target for enumeration

^b^ qPCR data with a 0.0 95% CI indicates either 1 or 0 of the duplicates provided a Cq value

^c^ No MPN was performed for this sample
