# Supplementary material for: Comparison of methods for the enumeration of enterohemorrhagic Escherichia coli from veal hides and carcasses
Source: Front Microbiol. 2015 Sep 29;6:1062. doi: 10.3389/fmicb.2015.01062 (PMC4586433; doi:10.3389/fmicb.2015.01062)
Supplement: Supplementary file 3 [file DataSheet1.DOCX]

***Supplementary Material***

**Comparison of methods for the enumeration of enterohemorrhagic *Escherichia coli* from veal hides and carcasses**

**Brandon E. Luedtke and Joseph M. Bosilevac^*^**

*** Correspondence:** Joseph M. Bosilevac, U. S. Department of Agriculture, Agricultural Research Service, Roman L. Hruska U. S. Meat Animal Research Center, State Spur 18D, Clay Center, Nebraska 68933-0166, USA, E‑mail: [mick.bosilevac@ars.usda.gov](mailto:mick.bosilevac@ars.usda.gov).





**Supplementary Figure 1: Five log standard curve for *eae* and *ecf1* for comparison to dPCR.** A five log standard curve of the EDL 932 gDNA was loaded in triplicate. The Cq values for *eae* and *ecf1* at each dilution were plotted against the expected concentration.
